# Nursing care for people with Chagas disease: a scoping review

**DOI:** 10.15649/cuidarte.3420

**Published:** 2024-05-29

**Authors:** José Antonio da, Álvaro Micael Duarte Fonseca, Micássio Fernandes de Andrade, Cléber de Mesquita Andrade, Ísis Kelly dos Santos, Ellany Gurgel Cosme do Nascimento

**Affiliations:** 1 Universidade do Estado do Rio Grande do Norte, Mossoró, Brazil. antoniodasilva@alu.uern.br Universidade do Estado do Rio Grande do Norte Universidade do Estado do Rio Grande do Norte Mossoró Brazil antoniodasilva@alu.uern.br; 2 Universidade do Estado do Rio Grande do Norte, Mossoró, Brazil. alv.micael@gmail.com Universidade do Estado do Rio Grande do Norte Universidade do Estado do Rio Grande do Norte Mossoró Brazil alv.micael@gmail.com; 3 Universidade do Estado do Rio Grande do Norte, Mossoró, Brazil. micassioandrade@uern.br Universidade do Estado do Rio Grande do Norte Universidade do Estado do Rio Grande do Norte Mossoró Brazil micassioandrade@uern.br; 4 Universidade do Estado do Rio Grande do Norte, Mossoró, Brazil. clebermesquita@uern.br Universidade do Estado do Rio Grande do Norte Universidade do Estado do Rio Grande do Norte Mossoró Brazil clebermesquita@uern.br; 5 Universidade do Estado do Rio Grande do Norte, Mossoró, Brazil. isiskelly@uern.br Universidade do Estado do Rio Grande do Norte Universidade do Estado do Rio Grande do Norte Mossoró Brazil isiskelly@uern.br; 6 Universidade do Estado do Rio Grande do Norte, Mossoró, Brazil. ellanygurgel@uern.br Universidade do Estado do Rio Grande do Norte Universidade do Estado do Rio Grande do Norte Mossoró Brazil ellanygurgel@uern.br

**Keywords:** Nursing Care, Chronic Disease, Communicable Diseases, Chagas Disease, Atención de Enfermería, Enfermedad Crónica, Enfermedades Transmisibles, Enfermedad de Chagas, Cuidados de Enfermagem, Doença Crônica, Doenças Transmissíveis, Doença de Chagas

## Abstract

**Introduction:**

Chagas disease is an infectious disease caused by the parasitism process of the protozoan *Trypanosoma cruzi.* Given its potential for chronicity, nursing care in the health care of patients with Chagas disease will provide an improvement in quality of life and the prognosis of the disease.

**Objective:**

Review scientific knowledge about nursing care for individuals with Chagas disease.

**Material and Methods:**

Descriptive and exploratory research, carried out with two independent reviewers using high sensitivity criteria in databases and gray literature sources between June and July 2022.

**Results:**

The review identified 12 relevant publications that emphasized health care, education, relationships, disease prevention and health promotion. The most frequent and diverse nursing diagnoses were related to the Activity/Rest, Health Promotion and Coping/Stress domains.

**Discussion:**

To meet the care needs of Chagas disease, it is essential to ensure nursing care that recognizes individualities, highlighting the importance of creating tools that facilitate the nursing process. The main points highlighted were related to the health education process, longitudinal monitoring, healthy lifestyle habits, general nursing care during hospitalization and the use of nursing diagnoses.

**Conclusion:**

The need for comprehensive nursing care that meets the main needs of individuals with Chagas disease is emphasized, considering their unique circumstances. Developing tools to support the nursing process is essential to improve the results of care for this population.

## Resumen

**Introducción:**

La enfermedad de Chagas es una enfermedad infecciosa causada por el proceso de parasitación del protozoo *Trypanosoma cruzi.* Dado el potencial de cronicidad de esta enfermedad, los cuidados de enfermería de pacientes con enfermedad de Chagas proporcionarán una mejora en la calidad de vida y en el pronóstico de esta enfermedad.

**Objetivo:**

Revisar los conocimientos científicossobrecuidados de enfermeríadirigidos a personascon enfermedad de Chagas.

**Materiales y Métodos:**

Investigación exploratoria y descriptiva que incluyó dos revisores independientes que aplicaron criterios de alta sensibilidad en las bases de datos y fuentes de literatura gris entre junio y julio de 2022.

**Resultados:**

La revisión identificó 12 publicaciones relevantes que hacen énfasis en la atención, la educación, las relaciones, la prevención de la enfermedad y la promoción de la salud. Los diagnósticos de enfermería más frecuentes y diversos estaban relacionados con los ámbitos actividad/descanso, promoción de la salud y afrontamiento/estrés.

**Discusión:**

Para atender las necesidades de cuidado de la enfermedad de Chagas, es fundamental garantizar cuidados de enfermería que reconozcan las características individuales, destacando la importancia de crear herramientas que faciliten el proceso de enfermería. Los principales puntos destacados estuvieron relacionados con el proceso de educación en salud, el seguimiento longitudinal, los hábitos de vida saludables, los cuidados generales de enfermería durante la hospitalización y el uso de diagnósticos de enfermería.

**Conclusión:**

Se hace énfasis en la necesidad de cuidados integrales de enfermería que atiendan las principales necesidades de las personas con enfermedad de Chagas, considerando sus circunstancias únicas. Es esencial el desarrollo de herramientas de apoyo al proceso de enfermería para mejorar los resultados de la atención a esta población.

## Resumo

**Introdução:**

A doença de Chagas é uma doença infecciosa causada pelo processo de parasitismo do protozoário *Trypanosoma Cruzi.* Dado o seu potencial de cronicidade, o cuidado de enfermagem na assistência à saúde dos pacientes com doença de Chagas proporcionará melhora na qualidade de vida e no prognóstico da doença.

**Objetivo:**

Revisar o conhecimento científico sobre os cuidados de enfermagem aos indivíduos com doença de Chagas.

**Material e Métodos:**

Pesquisa descritiva e exploratória, realizada com dois revisores independentes utilizando critérios de alta sensibilidade em bases de dados e fontes de literatura cinzenta entre junho e julho de 2022.

**Resultados:**

A revisão identificou 12 publicações relevantes que enfatizavam cuidados de saúde, educação, relacionamentos, prevenção de doenças e promoção da saúde. Os diagnósticos de enfermagem mais frequentes e diversos estavam relacionados aos domínios Atividade/Repouso, Promoção da Saúde e Enfrentamento/Estresse.

**Discussão:**

Para atender às necessidades de cuidado da doença de Chagas é fundamental garantir uma assistência de enfermagem que reconheça as individualidades, destacando a importância da criação de ferramentas que facilitem o processo de enfermagem. Os principais pontos destacados foram relacionados ao processo de educação em saúde, acompanhamento longitudinal, hábitos de vida saudáveis, cuidados gerais de enfermagem durante a internação e utilização de diagnósticos de enfermagem.

**Conclusão:**

Enfatiza-se a necessidade de uma assistência de enfermagem integral que atenda às principais necessidades dos indivíduos com doença de Chagas, considerando suas circunstâncias singulares. Desenvolver ferramentas de apoio ao processo de enfermagem é essencial para melhorar os resultados do cuidado a essa população.

## Introduction

Chagas disease is an infectious and contagious disease caused by the process of parasitism of the protozoan Trypanosoma cruzi. Transmission can be oral, vector, congenital, or transfusional, among others, and the different species of triatomines are the vectors responsible for this transmission. It can be classified according to clinical manifestations as indeterminate, cardiac, digestive, and cardio digestive. Furthermore, it can be classified as an acute or chronic infection^1^.

The Global Burden data of disease (GBD), for the year 2019, showed a prevalence rate of 79.9 per 100,000 people, an incidence rate of 2.3 per 100,000 people, and a mortality rate of 0.1 per 100,000 people, in the world^2^. In Brazil, a systematic review with meta-analysis estimated that the year 2010 a total of 4,6 million people were infected with T. cruzi in the country^3^. However, the data are imprecise due to the difficulty in the case notification process, mainly in Brazil.

Because of its characteristic of chronicity, Chagas disease requires multi- and interprofessional health care with the aim of improving the quality of life of its patients^4^and nursing care in the health care of this group will provide a better prognosis of the disease. For this, the Nursing Process is an important tool for the implementation of the Systematization of Nursing Care to guide the care of nursing professionals^5^^,^^6^.

The application of Nursing Process in clinical practice can still be seen as something that hinders the work process of professionals, especially in Primary Health Care. Despite this, it is necessary to emphasize that the Systematization of Nursing Care tends to contribute to the integrality of the care provided to people when performed in a convenient way, including at this level of health care^7^.

Thus, through the importance of the Systematization of Nursing Care to guide the practices of professionals, especially in outpatient follow-up, and the scarcity in the literature of systematic scientific material that addresses nursing care for people with Chagas disease. This study aimed to compile and detail the findings on nursing care provided to people with Chagas disease.

## Materials and Methods

This is a Scoping Review conducted following JBI scoping review orientations. The Protocol for this Scope Review has been registered with the Open Science Framework (OSF) with the following registration DOI: https://doi.org/10.17605/OSF.IO/W3VUJ.The acronym PCC was used for identification, namely: people with Chagas disease (Population), nursing care (Context), and nursing care (Concept). For this, the following guiding question was drawn up: How is care provided in nursing care for people with Chagas disease? The Dataset of this research is available at Mendeley Data^8^.

### Search strategy

The search was carried out in the following databases: Medical Literature databases Analysis and Retrieval System Online (Medline/ PubMed), Latin American and Caribbean Literature in Health Sciences (Lilacs /BVS), Embase, Cumulative Index to Nursing and Allied Health Literature (CINAHL), Database in Nursing (BDENF) and Web of Science.

To carry out a systematic search in these databases, the descriptors "Chagas Disease", "Nursing Care", "Self Care", "Models, Nursing" and some of their synonyms, were selected from Medical Subject Headings (MESH). In the Embase database, the descriptor “Models, Nursing” was replaced by the descriptor 'nursing theory', according to Emtree. To associate these terms, the Boolean operators AND and OR were used.

### Eligibility criteria

This review included scientific materials dealing with nursing care for people living with Chagas disease in written format with no time limit, language, age group, and/or type of health service. Materials that could not answer the guiding question of the research and those that were duplicated in the databases were excluded. For the exclusion step of duplicate articles, Zotero® software was used. As for the process of selecting articles by titles and abstracts, the Rayyan® application was used, after excluding duplicates.

### Data extraction

After this process, the materials were read in full. An instrument was used to collect data in the articles, extracting items related to the title, authors' names, research location, year of publication, study method, and main results of the studies. In addition, the material selection process was blindly carried out by two evaluators, with third and fourth evaluators being consulted in case of doubts about their inclusion.

It is emphasized the maintenance of the ethical character throughout the process of this research, aiming at the proper citations and references for the studies included in this Scope Review. Given this research method, it was not necessary to submit the project to the Research Ethics Committee.

## Results

**Figure 1:**
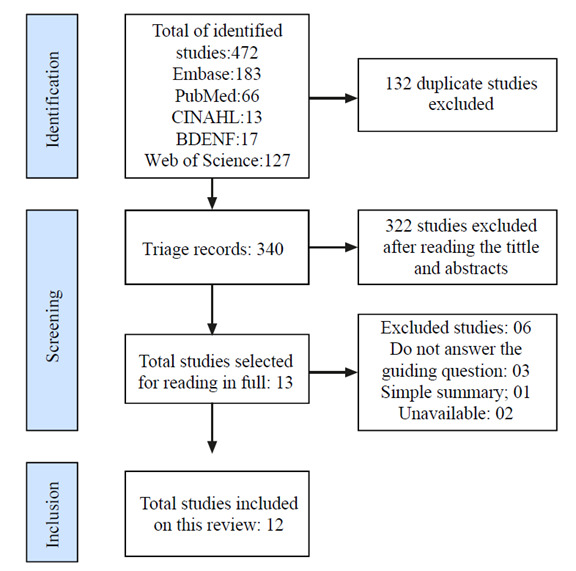
Flowchart with the number of articles in the review. Mossoró, Rio Grande do Norte, Brazil, 2022

Figure 1 describes the processes of identification, screening, and inclusion of the records found in the cited databases and of the included studies. For articles that were excluded due to unavailability, several ways were used to recover them on the Internet, all of which were unsuccessful.

A total of 12 publications, published between 1987 and 2019, mainly in the Americas, with emphasis on Latin American countries (n=10), were included in this study. There was a greater representation of experience report-type studies (25.00%), narrative review (16.67%), and descriptive and quantitative studies (16.67%). In total, there were 10 original articles, in addition to the fact that most of the works were constructed from an outpatient (25.00%) and hospital (25.00%) perspective, as shown in Table 1.

Among the studies that worked with nursing diagnoses for people with Chagas disease in the most diverse situations and age groups, they are contained in Figure 1. The domains were not described in the cited studies but were organized according to the domains of the 12th edition of the Diagnoses of Nursing at NANDA-I. The domains with the highest frequency and diversity of Nursing Diagnoses were Activity/rest (Domain 4), Health Promotion (Domain 1), and Coping/stress tolerance (Domain 9), respectively. In the publications that made up this study, there was no representation of the Life Principles domains (Domain 10), Comfort (Domain 12), and Growth/development (Domain 13) (Table 2).

**Table 1:** Description of methods, types of study, and level of health care of articles included in the literature review. Mossoró, Rio Grande do Norte, Brazil, 2022

Variables	n (%)
Country	
Brazil	08 (66.67)
Bolivia	01 (8.33)
Mexico	01 (8.33)
Canada	01 (8.33)
United States of America and Liberia	01 (8.33)
Study method	
Experience report	03 (25.00)
Case study	01 (8.33)
Revision narrative	02 (16.67)
Case-control	01 (8.33)
Descriptive and exploratory with a qualitative approach	01 (8.33)
Descriptive and exploratory with a quantitative approach	02 (16.67)
Search bibliographical	01 (8.33)
Revision Systematics of Literature	01 (8.33)
Type of study	
Completion of coursework	01 (8.33)
Original article	10 (83.33)
Guide informative	01 (8.33)
Level of healthcare	
Outpatient	03 (25.00)
Home	02 (16.67)
Hospital/hospitalization	03 (25.00)
All you levels	01 (8.33)
No description	03 (25.00)

**Table 2:** Diagnoses outlined for people with ChD from some of the studies. Mossoró , Rio Grande do Norte, Brazil , 2022

Domains/nursing diagnoses
Domain 1 - Health Promotion
Leisure activity deficit^9c^
Changing health maintenance^9c^
Effective control of the therapeutic regimen^10a^
Lack of adherence to treatment^9c^
Domain 2 - Nutrition
Altered nutrition less than body requirements / Nutritional deficit ^9c, 11b^
Swallowing impairment^9c^
Potential for excess water volume^9c^
Domain 3 - Elimination and exchange
Risk for constipation^11b^
Changing the pattern of urinary elimination^9c^
Altered intestinal elimination: constipation^9c^
Domain 4 - Activity/rest
Potential for decreased cardiac output^9c^
The deficit for self-care (hygiene, clothing, eliminations) (Level II)^9c^
Potential for activity intolerance^9c^
Sleep pattern disorder^9c^
Decreased physical mobility (Level III)^9c^
Level III activity intolerance^9c^
Domain 5- Perception/cognition
Deficient knowledge/Deficit of knowledge/Deficit of knowledge of the disease^9,11 a,b,c^
Domain 6 - Self-perception
Disturbance in body image due to the treatment of the disease^10a^
Change in self-esteem^9c^
Domain 7 - Roles and relationships
Impaired social interaction / Social isolation^9,10 a,c^
Changing paper performance^9c^
Domain 8- Sexuality
Changing patterns of sexuality^9c^
Domain 9 - Stress coping/tolerance
Anxiety^11b^
Fear^9,11 bc^
Adaptation impaired^9c^
Ineffective stress response (individual)^9c^
Domain 10 - Principles of life
Domain 11 - Security/protection
Potential for infection^9c^
Risk for impaired skin integrity/ Potential for skin injury^9,11 bc^
Hyperthermia^11b^
Domain 12 - Comfort
Domain 13 - Growth/development

^a^ Chagas disease with digestive involvement after colostomy surgery / ^b^ Hypothetical cases for generic cases / ^c^ Cases of hospitalization for chronic Chagas heart disease

**Figure 2:**
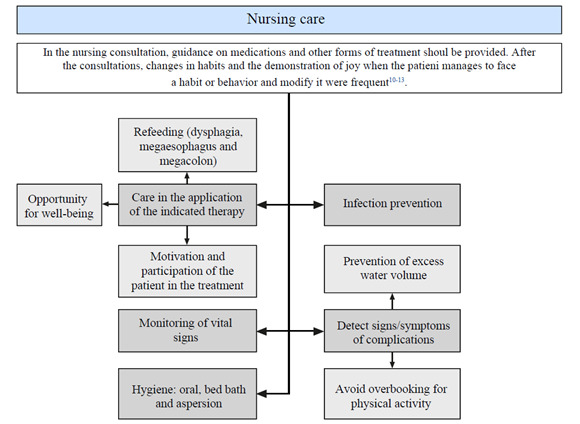
Description of the main results found in studies on nursing care provided to people with Chagas disease. Mossoró, Rio Grande do Norte, Brazil, 2022

It was observed that assistance through the nursing process has the role of developing care concerning Chagas disease in a more effective way. It is a care tool in various aspects of the life of the person with Chagas disease, such as maintaining their well-being and taking care of the signs and symptoms of the disease (Figure 2).

**Figure 3:**
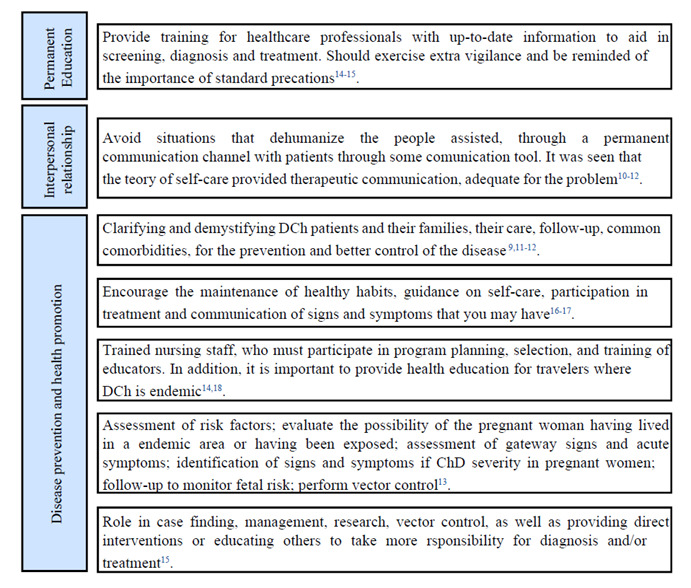
Description of the transversal line of care provided to people with Chagas disease. Mossoró, Rio Grande do Norte, Brazil, 2022

Other areas of nursing activity in Chagas disease were identified, being a cross-cutting line of care for this, but also other, more diverse professions in the health area. This cross-sectional line of care aims at the qualified training of health professionals, the interpersonal relationship between nurses and health service users, and the prevention of complications and injuries due to Chagas disease. In this perspective of disease prevention and health promotion, an important tool in health education both for people living with Chagas disease and for their families, caregivers, and the general population (Figure 3).

All studies included in this review brought examples of interventions in professional nursing practice that can be performed for people with Chagas disease, at the hospital and outpatient levels.

The searches carried out in the gray literature databases (Catalog of Theses and Dissertations by CAPES and ProQuest) did not bring any result of materials that answered the guiding question of this research, noticeable the scarcity of material from books, dissertations and specific theses on the theme studied.

## Discussion

The main points highlighted by the selected studies on nursing care for people with Chagas disease were related to the health education process, longitudinal follow-up, healthy living habits, general nursing care during hospitalization, and the use of nursing diagnoses. Faced with the diversity of possible conditions presented by people living with Chagas disease, the line of care must be broad and efficient, to identify the complications of the disease and the care prescribed for each case, aiming at preventing disability and rehabilitating these conditions. people, when necessary^19^.

In this sense, Chagas disease is described as endemic in Latin America, but there are already reports of new cases in countries considered non-endemic. Despite this, in this review, it was noticeable that the studies were from Latin American countries (Brazil, Mexico, Bolivia), probably because there is an endemic area. In health services, the studies permeate mainly the hospital and outpatient levels. From the perspective of preventive care, given that most people undergoing treatment have a stable clinical condition, Primary Health Care can play a differential role in the process of evolution of the signs and symptoms of Chagas disease^20^.

The lifestyle of people living with chronic diseases, such as Chagas disease, is a point of need for discussion, given that these people often need to adjust various aspects of their daily lives when receiving this type of diagnosis. In this sense, the findings concerning the NANDA-I domains of this study reflect this concern, considering that it has already been observed that people with Chagas heart disease have low lifestyle scores in the areas of physical activity, sleep, safety, stress, and safe sex^21^.

It should be noted that the practice of physical activity among people living with Chagas disease is an important tool for the prevention of intestinal signs and symptoms, which are often affected, because of the pathophysiological characteristics of Chagas disease^22^. It was also seen that physical training helps to improve the cardiac function of people with subclinical Chagas heart disease^23^.

Still in this panorama of style and quality of life, it is important to understand the aspects related to mental health and coping with the disease. In addition to the physical consequences, the emotional impact is notable, being linked to anxiety and fear of the disease and its prognosis. Even in asymptomatic cases, the news of the diagnosis can be traumatic for the people who receive it^24^.From the perspective of comprehensive care, the nursing consultation for people living with chronic diseases is a tool that already shows its role in monitoring these people, especially from the perspective of Primary Health Care^25^.

These findings guide how nursing care has been carried out for this specific public. Therefore, it was possible to observe little description of the main tools for the effective implementation of nursing care. Only one ofthe studies was able to describe the main nursing diagnoses for people with Chagas disease, which is an important gap in the scientific knowledge of the profession.

Nursing professionals play an important role in the health education process for the self-care of people who have some type of chronic disease^26^. It is no different for Chagas disease, considering that information, education and communication practices for health professionals and communities influence the improvement of testing and treatment demands, in addition to the quality of care provided to the population^27^. The results of this research also showed recognition of the importance of this patient's training process for the quality of nursing care.

Within the concept of transitional care in nursing, professionals see themselves as important actors in the adaptation process of patients and caregivers, in the case of elderly people in the process of rehabilitation and leaving hospitals, aiming at the continuity of care^28^. At the same time, a study carried out with coordinating nurses in the management of chronic diseases showed that it proved to be difficult to plan, develop and evaluate health education for the management of patients seen in primary services^29^.

Since Chagas disease is classified as a neglected disease, sociocultural factors are linked importantly in care, especially when talking about longitudinal. These barriers, especially in PHC, are not seen only in the reality of Brazilian health. These barriers consist of diagnostic difficulties, poor training of health professionals, and bureaucratic processes that make it difficult to treat the disease^30^.

This study signals the scarcity of scientific material that addresses nursing care, specifically for people with Chagas disease, highlighting mainly the Brazilian scientific literature. As it is an endemic disease in Brazil and Latin America, researchers in this geographical space must carry out investigations on the subject. In non-endemic countries for Chagas disease, health professionals seem to lack sufficient knowledge about the disease in question^31^.

So that nursing professionals can carry out this care process scientifically and efficiently, it is extremely important to use instruments that make this quality care process possible. The creation and use of nursing instruments specific to the reality of people with Chagas disease are important for the development of quality care, with adequate scientific knowledge^32^.

### Limitations:

As for the limitations for carrying out this study, they were due to the scarcity of more recent studies with more robust methodological designs, such as systematic reviews, meta-analysis, and clinical trials, in addition to being difficult to generalize due to their high specificity ofthe conditions studied.

## Conclusion

This scoping review identified some of the nursing care activities for people with Chagas disease, mainly in the hospital and outpatient settings. It was identified that the main care actions were related to the health education process, longitudinal follow-up, healthy living habits, and general nursing care during hospitalization. These findings contribute to the nursing team identifying the possibilities of interventions already practiced by other professionals in the nursing care provided to people with Chagas disease.

However, considering the lack of studies, the need for new studies aimed at identifying the main diagnoses and actions within nursing care for people with Chagas disease in a more inquired way and with more robust methodologies is highlighted. In addition, the importance of creating tools to facilitate the nursing process for this public is reiterated, through guiding instruments or even specific nursing diagnoses, results, and interventions, if necessary.
